# Complex stability and dynamic subunit interchange modulates the disparate activities of the yeast moonlighting proteins Hal3 and Vhs3

**DOI:** 10.1038/srep15774

**Published:** 2015-10-30

**Authors:** J. Albert Abrie, Cristina Molero, Joaquín Ariño, Erick Strauss

**Affiliations:** 1 Department of Biochemistry, Stellenbosch University, South Africa; 2 Institut de Biotecnologia i Biomedicina and Departament de Bioquímica i Biologia Molecular, Universitat Autònoma de Barcelona, Spain

## Abstract

*Saccharomyces cerevisiae* Hal3 and Vhs3 are moonlighting proteins, acting both as inhibitors of the serine/threonine protein phosphatase Ppz1 and as subunits (together with Cab3) of the unique heterotrimeric phosphopantothenoylcysteine decarboxylase (PPCDC) enzyme of Hemiascomycetous yeast. Both these roles are essential: PPCDC catalyses the third step of coenzyme A biosynthesis, while Ppz1 inhibition is required for regulation of monovalent cation homeostasis. However, the mechanisms by which these proteins’ disparate activities are regulated are not well understood. The PPCDC domains (PDs) of Hal3, Vhs3 and Cab3 constitute the minimum requirement for these proteins to show both PPCDC activity and, in the case of Hal3 and Vhs3, to bind to Ppz1. Using these PD proteins as a model system to study the possibility of dynamic interchange between these roles, we provide evidence that Hal3 binds Ppz1 as a monomer (1:1 stoichiometry), requiring it to de-oligomerize from its usual homo- and heterotrimeric states (the latter having PPCDC activity). This de-oligomerization is made possible by structural features that set Hal3 apart from Vhs3, increasing its ability to undergo monomer exchange. These findings suggest that oligomer interchange may be a significant factor in the functional regulation of these proteins and their various unrelated (moonlighting) functions.

Increasing numbers of moonlighting proteins—proteins that are able to perform multiple functions within a single polypeptide chain—are being discovered, calling for a paradigm shift in biology from the one protein, one function concept^1^,^2^,^3^. The apparent prevalence of moonlighting proteins adds an additional level of complexity to cellular physiology, and the regulation of the multiple functions of such proteins are not well understood^4^. In the case of moonlighting proteins that form part of multicomponent complexes, the relative stability of the complex could form the mechanistic basis whereby such regulation may be achieved, especially if dissociation from the complex is an essential requirement for the protein to fulfil its other physiological functions.

*Saccharomyces cerevisiae* Hal3 (also referred to as Sis2) and Vhs3 have been classified as moonlighting proteins based on their dual function in both regulatory and biosynthetic pathways (Fig. 1a)^5^. In regards to regulation, these proteins have been shown to individually act as inhibitors of the Ser/Thr phosphatase Ppz1, which plays a role in the regulation of monovalent cation homeostasis, which impacts on cell-cycle control, halotolerance and cell-integrity^6^,^7^,^8^,^9^. In terms of biosynthesis, Hal3, Vhs3 and Cab3 (previously known as Ykl088w) form the constituent parts of a unique heterotrimeric phosphopantothenoylcysteine decarboxylase (PPCDC) enzyme that catalyses the third step of the universal coenzyme A (CoA) biosynthetic pathway, namely the decarboxylation of 4′-phosphopantothenoylcysteine (*P*PC) to form 4′-phosphopantetheine (*P*PantSH) (Fig. 1b).

In most eukaryotes, as typified by the human (HsCoaC) and *Arabidopsis thaliana* (AtHal3a) proteins, PPCDC is a homotrimer with three active sites formed at the oligomer interaction interfaces^10^,^11^,^12^,^13^. Importantly, two catalytically essential residues—a His that is required for the first step of the enzyme’s two-step mechanism (an oxidative decarboxylation), and a Cys that is necessary for the second step (the reduction of the reaction intermediate)—are found on opposite sides of the active site, with each of the adjacent protomers donating one of these residues^14^. However, not one of the yeast proteins contains both catalytically essential residues. Instead, Hal3 and Vhs3 only contain the His, while Cab3 has the requisite Cys residue, in addition to a non-functional His^5^. Consequently, the homotrimeric versions of these proteins cannot and do not show any PPCDC activity. Instead, in the heterotrimeric protein a single active site is formed per trimer at the interface between adjacent Hal3/Vhs3 and Cab3 protomers (Fig. 1a).

Sequence analysis suggests that Hal3, Vhs3 and Cab3 are structurally related, exhibiting three distinct domains: an N-terminal domain without significant sequence homology outside of yeasts, a PPCDC domain (PD) with high sequence homology to known PPCDCs, and a highly acidic C-terminal tail. Functional mapping of the three domains of Hal3 revealed that the presence of its PD is the minimum requirement for PPCDC activity, although the N-terminal domain appears important in stabilizing interactions between Hal3 and Cab3^15^. The PD is also essential for binding to Ppz1, while the N- and C-terminal domains are required for Ppz1 inhibition^6^,^15^. Mutagenesis studies of Hal3’s PD indicated that some of the residues that are functionally important for Ppz1 inhibition are also associated with, although not essential for, PPCDC function^5^,^6^. Moreover, it still remained unknown whether Hal3 interacts with Ppz1 as a monomer or as a trimer.

The studies described above raise several questions about the mechanism regulating the dual functions of Hal3 and Vhs3. Specifically: does the Hal3/Vhs3/Cab3 heterotrimer represent the major cellular pool from which these proteins dissociate to exert their Ppz1 inhibitory functions? If so, this would require the formation of PPCDC heterotrimers to be a dynamic process. However, if they are stable once formed, would that indicate that a heterogenous population is present that consists of heterotrimers acting as PPCDC enzymes, and more labile Hal3 and Vhs3 homotrimers that serve the requirement for Ppz1 inhibitors? In the light of our current lack of understanding of the regulatory mechanisms of moonlighting proteins in general, these questions hold special interest as the Hal3/Vhs3/Cab3 oligomerisation dynamic could form the basis for the manner in which Hal3 and Vhs3’s relative participation in CoA biosynthesis compared to cellular regulation is controlled.

In this study we set out to find answers to these questions, first by defining the stoichiometry of Ppz1 and Hal3 interaction and then by studying the interaction of PDs of Hal3, Vhs3 and Cab3. Our use of the PDs as a model system was based on previous observations that the Hal3 PD is sufficient for both its PPCDC function and its ability to bind to Ppz1^15^. Additionally, the available structural data of known homotrimeric PPCDCs—such as HsCoaC and AtHal3a^10^,^11^—and the high sequence homology of the Hal3, Vhs3 and Cab3 PDs to these known PPCDCs, suggest that the PD is primarily responsible for determining the oligomerisation state and PPCDC activity of these proteins (and their complexes). We therefore investigated the Hal3, Vhs3 and Cab3 PD proteins by a variety of biochemical and biophysical techniques including PPCDC activity assays, size exclusion chromatography (SEC) and circular dichroism (CD). Our results show that there are significant structural differences between Hal3 and Vhs3, and that oligomeric mixtures made up of these proteins exhibit different thermal stabilities based on their composition, as well as varying abilities to undergo monomer exchange. These findings point to the dynamic interchange of oligomer subunits as a significant factor in the functional regulation of these proteins’ moonlighting functions.

## Results

### Elucidation of the Ppz1-Hal3 complex stoichiometry

The composition of the Ppz1-Hal3 complex was initially investigated in a pull-down assay by passing Hal3 (obtained from GST-Hal3 by PreScission protease treatment) over GST-Ppz1^Cter^ bound to glutathione-agarose beads. The use of Ppz1^Cter^ instead of the entire Ppz1 protein was based on the observation that its binding to Hal3 is facilitated by removal of the Ppz1 N-terminal extension, which likely acts as a protective domain^1^,^9^,^16^. Diverse Ppz1^Cter^/Hal3 ratios were used, ranging from 0.5-fold to a 6-fold excess of Hal3, followed by washing off unbound Hal3 and recovery of the Ppz1-Hal3 complex. The complex was analysed by SDS-PAGE to evaluate the relative amounts of Hal3 and Ppz1 (Fig. 2a). Even in the presence of a substantial excess of Hal3 in the mixture, the Hal3/Ppz1^Cter^ ratio did not even reach unity, instead showing a maximum of around 0.5 (Fig. 2b). This suggests that the binding conditions may be non-optimal, possibly due to steric effects caused by the binding of GST-Ppz1^Cter^ to the beads.

To further investigate the nature of the complex, we attempted its co-purification. For this purpose we co-expressed an N-terminally 6 × His-tagged version of Ppz1^Cter^ and an untagged version of Hal3 from the plasmid pETDuet-1 which has two multiple cloning sites, each with their own promoter and ribosome binding site. The Ppz1^Cter^–Hal3 complex was purified by immobilized metal affinity chromatography (IMAC) using Ni^2+^-based affinity resins and concentrated. When this sample was subjected to SEC a single major peak with an apparent molecular weight of ~165 kDa was recovered (Fig. 2c). SDS-PAGE analysis of the column fractions (Fig. 2d) revealed that the peak contained Hal3 and Ppz1^Cter^ in a ratio of nearly 1:1 (based on densitometric analysis and correcting for the relative fraction of residues known to bind Coomassie-Blue). While the apparent complex size is larger than the expected size for a 1:1 complex of Hal3 and Ppz1 monomers (~100 kDa), it is smaller than alternative 1:1 complexes (eg. two or three subunits of each protein). It is important to note that we found that purified Hal3 also elutes on SEC with an apparent size substantially larger (~104 kDa, not shown) than its actual MW (64 kDa), most likely due to the non-globular nature of its N- and C-terminal domains. Consequently, its presence in the Ppz1^Cter^–Hal3 complex is probably responsible for the anomalous elution behaviour of this combination.

Taken together, our data strongly suggest a 1:1 binding interaction for Hal3 and Ppz1, supporting the hypothesis that the Hal3 monomer (and not its previously characterized homotrimeric version^5^,^15^) acts as a Ppz1 inhibitor. This notion is further supported by the previous observation that the combination of equivalent amounts of Hal3 and Ppz1^Cter^ result in near complete inhibition of the latter’s phosphatase activity^7^,^9^,^17^. This finding has far-reaching implications, as it indicates that Hal3 must abandon its role as a member of the heterotrimeric yeast PPCDC or dissociate from its homotrimeric form through de-oligomerization to fulfil its role as inhibitor of Ppz1. To investigate this further, we set out to prepare the PD versions of the constituent members of the yeast PPCDC to determine if these proteins could act as suitable models to study this phenomenon.

### Expression and purification of the PDs of Hal3, Vhs3 and Cab3

The PDs of Hal3 (Hal3^PD^) and Vhs3 (Vhs3^PD^) were successfully expressed in high yield as N-terminal 6 × His-fusions from cultures transformed with the relevant plasmids. The expression of the PD of Cab3 (Cab3^PD^) did, however, present greater difficulty. Numerous attempts to express Cab3^PD^ by varying temperature and IPTG induction concentrations, or by using expression plasmids with different promoters (T7 and trc-based) or that encode different fusion tags (i.e. 6 × His, glutathione *S*-transferase, NusA and maltose binding protein) did not result in in the production of purifiable protein (data not shown). This suggests that the PD of Cab3 is either not successfully translated or immediately degraded upon expression in the heterologous host.

We next attempted to prepare Cab3^PD^ through the same co-expression strategy used to study the Hal3/Ppz1^Cter^ complex. To this end untagged Cab3^PD^ was co-expressed with either 6 × His-Hal3^PD^ or 6 × His-Vhs3^PD^ using the same pETDuet-1 system, described above. Gratifyingly, Cab3^PD^ was found to give significant expression in both cases, although considerably more total protein was obtained from co-expression with 6 × His-Hal3^PD^ than with 6 × His-Vhs3^PD^. We were able to co-express all three proteins by co-transforming a plasmid encoding Vhs3^PD^ with the plasmid expressing both Hal3^PD^ and Cab3^PD^.

The expressed proteins were subsequently purified using Ni^2+^-based IMAC. SDS-PAGE analysis (Fig. 3a) shows that the untagged Cab3^PD^ that was co-expressed with the His-tagged Hal3^PD^ or Vhs3^PD^ (either separately, or in combination) co-purifies with the tagged proteins, providing strong evidence that it forms a strong interaction with both Hal3^PD^ and Vhs3^PD^. The difficulty that was experienced in expressing Cab3^PD^ in the absence of either of the other PD proteins is therefore most likely due to it requiring interaction with Hal3^PD^ or Vhs3^PD^ for structural stability. This analysis is supported by a recent report suggesting that Cab3’s ability to form stable homomeric complexes requires its N-terminal domain^18^.

Densitometric analysis of the bands corresponding to the purified proteins in the various mixtures also indicates that in the case of the two binary mixtures (Hal3^PD^/Cab3^PD^ and Vhs3^PD^/Cab3^PD^), Hal3^PD^ and Vhs3^PD^ is present in at least two-fold excess compared to Cab3, while for the ternary mixture the ratio of Hal3^PD^:Vhs3^PD^:Cab3^PD^ is approximately 2:1:1 (Fig. 3a). This would suggest that the minimal heteromeric complex formed in each case would be (Hal3^PD^)_2_/Cab3^PD^, (Vhs3^PD^)_2_/Cab3^PD^ and Hal3^PD^/Vhs3^PD^/Cab3^PD^ respectively, although the excess of Hal3^PD^ in the ternary mixture suggest that more than one type of complex might be present in this case (such as a combination of (Hal3^PD^)_2_/Cab3^PD^ and Hal3^PD^/Vhs3^PD^/Cab3^PD^).

### The PDs of Hal3, Vhs3 and Cab3 contain the structural elements required for PPCDC activity and trimerisation

To investigate the composition and identity of the PD complexes further, PPCDC activity and oligomer characterization studies were performed. The PD constructs exhibited a similar PPCDC activity profile (Fig. 3b) to that previously observed for the full-length proteins^5^, confirming that an active complex is formed in the presence of Cab3^PD^ and either Hal3^PD^ or Vhs3^PD^. This indicates that the PDs of these proteins are sufficient for PPCDC activity as expected from sequence homology studies. Furthermore, the observation of activity suggests that the PDs also contains the structural elements required for trimerisation, as the active sites of the yeast PPCDC are found at the interface between adjacent monomers and are therefore only properly constituted in the trimeric protein^5^. This result also confirms our previous observation that the Hal3^PD^ can interact with the full-length Cab3 to yield a protein complex with PPCDC activity^15^. We observed similar levels of activity with the different Cab3^PD^ containing mixtures, which contrasts with the results obtained with the full length proteins where the Vhs3/Cab3 heterotrimer showed significantly reduced activity^5^. The comparable activity levels of Hal3^PD^/Cab3^PD^ and Vhs3^PD^/Cab3^PD^ could suggest increased stability of the latter complex compared to its full-length equivalent.

The ability of the PDs to form oligomers was investigated in more detail by means of cross-linking studies and SEC. Chemical cross-linking experiments were performed with disuccinimydyl suberate (DSS), a compound that reacts with primary amine groups (including lysine side-chains and the N-termini of polypeptides) to form amide bonds. SDS-PAGE analyses of DSS-treated 6 × His-Hal3^PD^ (Fig. 4a), with a monomeric molecular weight (MW) of 29.1 kDa, yields two species with MWs of 50–60 kDa and 85–100 kDa respectively, which corresponds well with the expected MWs of dimers (58.2 kDa) and trimers (87.3 kDa). Similar results were obtained with 6 × His-Vhs3^PD^, which has a monomeric MW of 34.5 kDa. Two species with MWs of 60–70 kDa and 100–120 kDa are observed by SDS-PAGE, corresponding to the expected respective molecular weights of dimers (69 kDa) and trimers (103.5 kDa). The observed dimers are most likely formed due to incomplete cross-linking.

SEC studies of the Hal3^PD^ and Vhs3^PD^ proteins (Fig. 4c,d) and the Hal3^PD^/Cab3^PD^ and the Hal3^PD^/Vhs3^PD^/Cab3^PD^ mixtures (Fig. 4e,f) support the conclusions drawn from the cross-linking results (the Vhs3^PD^/Cab3^PD^ mixture was not included in this analysis, due to the low expression levels for this complex limiting the amount of protein available for analysis). For these studies, the human PPCDC HsCoaC, which has previously been shown to form a trimer, was used as positive control (Fig. 4b). In all cases the MW of the major oligomeric species corresponded to the MW of the expected trimeric complex, except for the Hal3^PD^/Cab3^PD^ complex (Fig. 4e), where an apparent hexameric (or dimer of trimers) species predominates. Hal3^PD^ (Fig. 4c) and the HsCoaC control (Fig. 4b) were also found to form higher oligomeric structures, including hexamers and dodecamers, while in the case of Vhs3^PD^ (Fig. 4d) and the Hal3^PD^/Vhs3^PD^/Cab3^PD^ mixture (Fig. 4f) only trimeric complexes were observed. While the ternary mixture only shows a single peak that corresponds closely with the expected MW of the Hal3^PD^/Vhs3^PD^/Cab3^PD^ trimer, the low resolution of the SEC analysis makes it impossible to exclude the possibility that (Hal3^PD^)_2_/Cab3^PD^ trimers are present as well.

### Vhs3^PD^ differs from Hal3^PD^ based on secondary structure and mode of flavin binding

**Figure 1:**
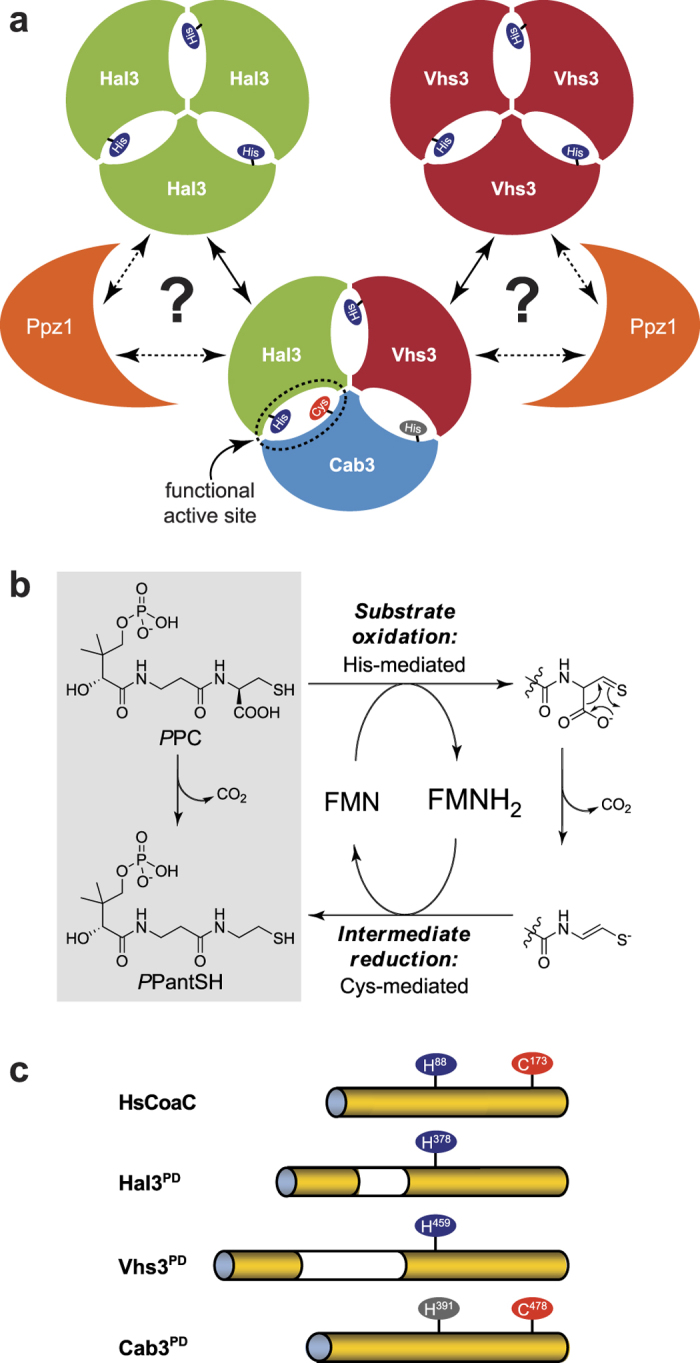
The yeast PPCDC enzyme and its constituent proteins. (**a**) Schematic representation of the active PPCDC in yeast, its constituent components and the various questions surrounding their respective interactions. The heterotrimeric PPCDC is formed by the combination of Hal3 and/or Vhs3 (which contributes the catalytically essential His residue, shown in blue) with Cab3 (which contributes the catalytically essential Cys residue, shown in orange) to give a complex with a single functional active site. Note that although Cab3 also has a His residue (shown in grey), it has been shown to be non-functional^5^. In addition, both Hal3 and Vhs3 also exist as homomeric trimers, and act as inhibitors of the protein phosphatase Ppz1 (shown in orange). Characterizing the interchange of monomers between the homo- and heterotrimers and the nature of the inhibitory interaction with Ppz1 is the focus of this study. (**b**) The reaction catalysed by PPCDC enzymes (in grey box) with its two-step mechanism shown on the right. Note that the catalytically essential His residue is important for the first step (the oxidative decarboxylation), while the Cys is required for the reduction of the enethiol intermediate (the second step). (**c**) Schematic alignment of the PDs of Hal3, Vhs3 and Cab3 with human PPCDC (HsCoaC). The non-homologous insertion regions of Hal3^PD^ and Vhs3^PD^ are represented in white, while the catalytically essential His (in HsCoaC, Hal3^PD^ and Vhs3^PD^) and Cys (in HsCoaC and Cab3^PD^) residues are indicated in blue and orange respectively. The non-functional His residue in Cab3^PD^ is denoted in grey.

**Figure 2:**
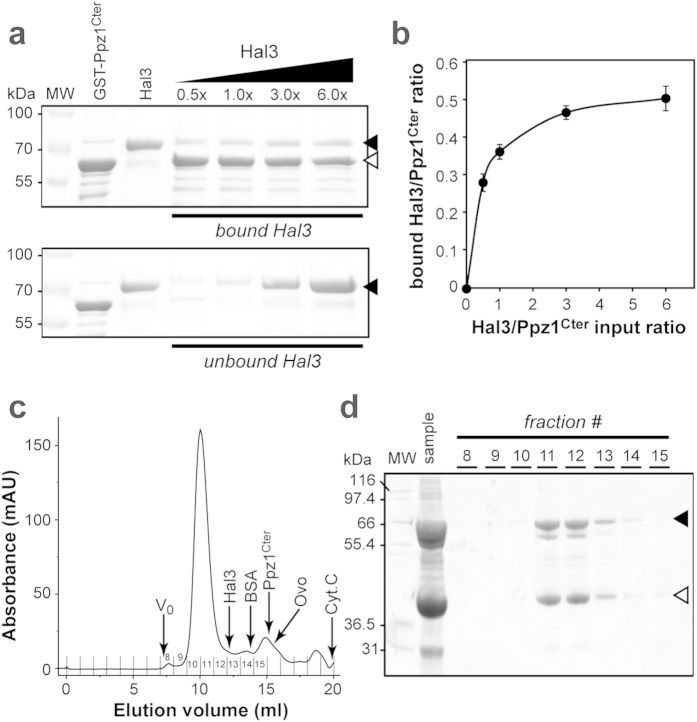
Defining the Ppz1/Hal3 complex stoichiometry. (**a**) Increasing amounts of recombinant Hal3 were incubated with GST-Ppz1^Cter^ bound to glutathione-agarose beads. After recovering the flow-through and washing, the beads were resuspended in Laemmli buffer, boiled, and aliquots (60 μL) of the supernatant analyzed by 8% SDS-PAGE followed by Coomassie staining. The upper panel shows the amounts of Hal3 bound by GST-Ppz1^Cter^, whereas the lower panel illustrates the amounts of unbound Hal3 present in the flow-through (40 μL samples). The first 2 lanes include samples (2 μg) of GST-Ppz1^Cter^ and Hal3 proteins used in the experiment (note that the band present in the GST-Ppz1^Cter^ sample that is similar in size to Hal3 is in fact the *E. coli* DnaK chaperone, as established by mass spectrometry analysis). Bands corresponding to Hal3 are identified with a closed arrow; GST- Ppz1^Cter^ with an open arrow. Molecular weight (MW) is in kilodalton (kDa). (**b**) The relative ratio bound Hal3/GST-Ppz1^Cter^ was obtained by scanning the stained gels and integrating the relevant signals with Un-Scan-It Gel (Silk Scientific) software. Data represents the mean ± SD from two independent experiments. (**c**) 6 × His-Ppz1^Cter^ and Hal3 were co-expressed from plasmid pETDUET-1, the complex recovered by Ni^2+^-affinity chromatography and concentrated (“sample” in panel D). The sample was subjected to SEC (Superdex 200) and the shown A_280_ elution profile was recorded. Arrows indicated the void volume (V_0_) and the elution volumes (V_e_) of known proteins: bovine serum albumin (BSA, 67 kDa), ovalbumin (Ovo, 43 kDa) and cytochrome C (Cyt.C, 12.3 kDa). The elution volumes of recombinant Ppz1^Cter^ (42.3 kDa) and Hal3 (64 kDa) are also shown; note that the latter elutes with a V_e_ equivalent to that of a ~104 kDa protein. The numbers denote the fractions collected. (**d**) The indicated fractions (1 mL) in panel C were collected; 10 μL of each was subjected to 10% SDS-PAGE analysis and stained with Instant Blue (Expedeon). Note the similar intensity of the bands at ∼66 kDa (Hal3) (◀) and ∼40 kDa (6 × His-Ppz1^Cter^) (◁), indicating a 1:1 protein ratio in the eluted complex. MW, molecular weight markers.

Having established that the PD proteins reflect the same activity and oligomerization characteristics as their native counterparts, we next set out to study their structural features as possible basis for the modulation of their oligomer stability. The secondary structure of the various PD domains and their corresponding complexes were studied by CD spectroscopy in the far-UV range. The obtained CD spectra (Fig. 5a) suggest that the proteins differ in secondary structure, most likely due to differences in the non-homologous inserts found in Hal3 and Vhs3 as the proteins share a high level of sequence similarity in other regions (Fig. 1c). Oligomers of Hal3^PD^ as well as the Vhs3^PD^/Cab3^PD^ mixture are primarily α-helical as indicated by the absorbance bands at 208 and 222 nm. Vhs3^PD^ appears to be structurally different from Hal3^PD^ and other typical α-helical proteins due to the greater intensity of the 208 nm band relative to the 222 nm band in its case. Furthermore, introduction of Cab3^PD^ into the complex affects the global secondary structure as a decrease in the intensity of the 208 nm band is observed in the Hal3^PD^/Vhs3^PD^/Cab3^PD^ and Hal3^PD^ /Cab3^PD^ in comparison to Hal3^PD^ alone, while a decrease in the 222 nm band is observed in the case of Vhs3^PD^/Cab3^PD^ in comparison to Vhs3^PD^ only. This suggests that Cab3^PD^ is structurally different from both Hal3^PD^ and Vhs3^PD^, and/or that the introduction of Cab3^PD^ into the oligomers results in conformational changes in the proteins involved in the formed heterotrimer.

The CD spectra of flavoproteins in the visible range provide a sensitive indication of differences in these proteins’ binding of their flavin cofactor^19^. Since the flavins of PD proteins are bound in the active sites that are found at the trimer interfaces, analysis of the CD and UV-visible absorption spectra of these proteins should provide an indication of the differences and similarities of their respective subunit interactions. The flavin CD spectra of the purified PD proteins and the various mixtures were therefore obtained (Fig. 5b). Although these spectra overall show a high level of similarity, in the case of Vhs3^PD^ a slight red shift can be observed for the absorption band that is found at ~375 nm in the other proteins. The slight red shift is also observed in the absorbance maximum of the ~375 nm peak in the corresponding absorbance spectrum (obtained simultaneously with the CD spectra) of Vhs3^PD^ compared to those of the other proteins and mixtures (Fig. 5c). An additional low intensity absorption band is also visible at ~465 nm in the Vhs3^PD^ flavin CD spectrum and in those of the heteromeric complexes containing Vhs3^PD^ (Fig. 5b). These differences suggest that the intersubunit interactions in oligomers that contain Vhs3^PD^ are dissimilar from those found in Hal3^PD^, either on its own or in complex with Cab3^PD^.

### Heat-induced secondary structure changes reveal differences in the thermal stability of the trimeric complexes

Oligomer thermal stability based on changes in secondary structure was investigated by studying changes in the CD signal at 222 nm—the wavelength normally used for studying proteins that largely consist of α-helices^20^—as a function of temperature. The obtained data were converted to represent the fraction of folded protein (see Experimental for details), which gave the protein melting curves represented in Fig. 6a. The corresponding first derivative plots of these melt curves are given in Fig. 6b; these were used to estimate the melting temperatures (T_m_) in each case, as many of the obtained curves exhibited multiple inflection points (suggesting several separate structural changes) that prevented a single equation from being fitted to the data. Importantly, protein unfolding was found to occur irreversibly due to protein precipitation, precluding the calculation of thermodynamic constants from the obtained data^21^. The various T_m_-values obtained in this manner are summarised in Table 1.

Comparison of these values indicate that Hal3^PD^ and Vhs3^PD^ exhibit dramatically different stabilities as demonstrated by their T_m_-values of ~45 ^o^C and 78 ^o^C respectively, which correspond to the lowest and highest values determined for any of the proteins and mixtures. Vhs3^PD^ exhibited two T_m_s that are similar in value to the two T_m_s determined for HsCoaC (the known human PPCDC). However, in both cases the lower value is based on a low intensity peak in the first derivative curves (i.e. a minor change in the heat-induced melt curves, most likely associated with a minor structural change). In contrast three apparent T_m_-values can be determined for Hal3^PD^ (Fig. 6b), with the lowest of the three most probably being the functionally relevant value. This conclusion is based on the finding that the higher values correlate with a distinctive reduction in this protein’s absorbance (starting at ~55 °C) that is likely due to protein precipitation and the subsequent settling of the precipitate in the cuvette (Supplementary Fig. S1). Taken together, this indicates that Vhs3^PD^ forms the most stable protein complexes, while the Hal3^PD^ complexes are the least stable. Correspondingly, Cab3^PD^’s complexes with Vhs3^PD^ are much more stable than with Hal3^PD^; in fact, addition of Cab3^PD^ to homomeric Vhs3^PD^ lowered the latter’s major T_m_ by ~5 °C, while for Hal3^PD^ the presence of Cab3^PD^ had a stabilizing effect, increasing its T_m_ by ~10 °C. Predictably, the ternary Hal3^PD^/Vhs3^PD^/Cab3^PD^ complex showed intermediate stability compared to the two binary complexes.

**Figure 3:**
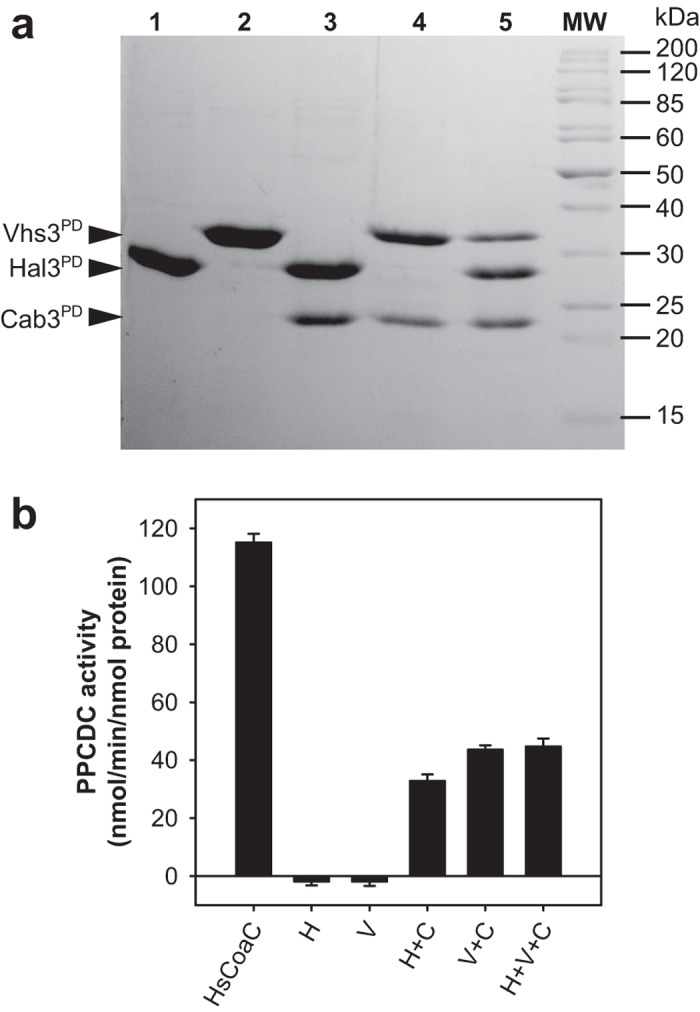
SDS-PAGE and PPCDC activity analyses of purified PD proteins. (**a**) SDS-PAGE analysis (using a 12% gel) of ~5 μg of each of the pure PD proteins or the indicated mixtures. Lane 1, 6 × His-Hal3^PD^ (expected MW: 29.1 kDa); lane 2, 6 × His-Vhs3^PD^ (expected MW: 34.5 kDa); lane 3, co-expressed 6 × His-Hal3^PD^/Cab3^PD^ (expected Cab3 MW: 23.3 kDa); lane 4, co-expressed 6 × His-Vhs3^PD^/Cab3^PD^; lane 5, co-expressed 6 × His-Hal3^PD^/6 × His-Vhs3^PD^/Cab3^PD^; MW, molecular weight markers with molecular weights indicated. (**b**) *In vitro* PPCDC activity of ~60 nM of either HsCoaC, Hal3^PD^ (H), Vhs3^PD^ (V), Hal3^PD^/Cab3^PD^ (H + C), Vhs3^PD^/Cab3^PD^ (V + C), and co-expressed Hal3^PD^/Vhs3^PD^/Cab3^PD^ (H + V + C). The data represent the average of three separate experiments, with the error bars showing the standard deviation.

The Hal3^PD^/Vhs3^PD^/Cab3^PD^-mixture exhibited two distinct peaks of similar intensity, which suggests that the complex formed in its case either undergoes two distinct temperature-induced structural changes, or that the mixture contains two different protein complexes. Since the SDS-PAGE analysis of the purified Hal3^PD^/Vhs3^PD^/Cab3^PD^ protein mixture indicated that that Hal3^PD^ is present in at least two-fold excess compared to the other two proteins (refer Fig. 3a), it is likely that this mixture contains two active heterotrimers, i.e. Hal3^PD^/Vhs3^PD^/Cab3^PD^ and (Hal3^PD^)_2_/Cab3^PD^. This analysis is supported by the finding that the Hal3^PD^/Cab3^PD^ mixture shows only a single T_m_ that matches the value of the lowest T_m_ observed for the Hal3^PD^/Vhs3^PD^/Cab3^PD^ mixture (Table 1), leading us to conclude that this value most likely represents the T_m_ of the (Hal3^PD^)_2_/Cab3^PD^ complex, while the higher value represents the true Hal3^PD^/Vhs3^PD^/Cab3^PD^ T_m_.

### Heat-induced flavin release by the trimeric complexes precedes denaturation in some cases

The finding that some the proteins and mixtures exhibit multiple apparent T_m_-values was not completely unexpected, since it is also possible that at least in some instances the trimeric proteins first dissociate into their constituent subunits, and that this is then followed by the major gross structural changes that lead to denaturation. Since the models of the trimeric PPCDC and related enzymes all indicate that the flavin binding site is located at the interface of the subunits (Fig. 1a)^10^,^11^, one would predict that dissociation of the oligomeric complexes would lead to exposure of the flavin cofactor to solvent and its release from the protein complex. Importantly, free flavin is known to exhibit low levels of ellipticity, while the binding of flavin to a protein significantly increases the intensity of its CD spectrum due to the restrictive and asymmetric nature of the binding site^19^. We therefore evaluated the heat-induced changes in the molar ellipticity of the various proteins’ flavin cofactors to determine whether dissociation of the protein complexes precedes or accompanies the unfolding/denaturation.

The flavin CD spectra of the PD proteins were found to show the largest heat-induced changes in molar ellipticity at ~320 nm (Supplementary Fig. S2); consequently, readings at this wavelength were obtained for each of the proteins and mixtures at increasing temperatures. The readings were converted to the fraction of protein-bound flavin, giving the plots shown in Fig. 6c. The respective T_m_-values (only one in each case) were obtained by fitting of the Gibbs-Helmholtz equation (see Experimental for details) (Table 1), and were found to follow the same trend as determined from the secondary structure changes. More specifically, the data confirm that Hal3^PD^ is the least stable and Vhs3^PD^ the most stable of the PD proteins, with complexes that contain Cab3^PD^ having intermediate stability. Moreover, comparison of the flavin release-based T_m_-values to the T_m_s obtained from the secondary structure-based changes indicated that in most instances flavin release (and therefore trimer dissociation) occurs first, followed by denaturation at higher temperatures. Vhs3^PD^ was the only protein for which the two values were found to coincide, indicating that release of its flavin cofactor requires unfolding and denaturation. It is therefore unlikely to exchange subunits with other trimeric complexes. In contrast, these findings suggest that subunit dissociation (and therefore subunit exchange) is a likely event in the case of Hal3^PD^ and the Hal3^PD^/Cab3^PD^ mixture. For the Hal3^PD^/Vhs3^PD^/Cab3^PD^ mixture our analysis of the secondary structure-based changes suggested that two complexes—i.e. Hal3^PD^/Vhs3^PD^/Cab3^PD^ and (Hal3^PD^)_2_/Cab3^PD^—are present. However, only a single flavin release-based T_m_-value is observed in its case. This would suggest that of the two only (Hal3^PD^)_2_/Cab3^PD^ is susceptible to subunit dissociation, or that the two complexes’ susceptibility to dissociation is indistinguishable.

### Heterotrimeric complexes exhibit different abilities to undergo subunit exchange

To further investigate the propensity of the heterotrimeric (Hal3^PD^)_2_/Cab3^PD^ and Hal3^PD^/Vhs3^PD^/Cab3^PD^ complexes to undergo monomeric subunit exchange, PPCDC inactive forms of the complexes were prepared by mutation of the catalytically essential His residue in either Hal3^PD^ or Vhs3^PD^ to alanine to form Hal3^PD_H378A^ and Vhs3^PD_H459A^ respectively. These complexes were subsequently titrated with either native Hal3^PD^ or Vhs3^PD^, incubated for 10 minutes, and then analysed for their PPCDC activity. We expected to only observe PPCDC activity in the instances that the native proteins displaced their mutated counterparts by dissociation and subunit exchange, with the relative magnitude of the PPCDC activity providing an indication of the extent of exchange.

For the Hal3^PD_H378A^/Cab3^PD^ mixture we observed a significant increase in the activity upon incubation with an excess of Hal3^PD^, indicating successful subunit exchange between the respective oligomers (Fig. 7). Similarly, we observed activity upon treatment of the same mixture with Vhs3^PD^, although in this case the observed increase in activity was markedly less than what was seen for Hal3^PD^. This is most likely caused by lower levels of exchange between Vhs3^PD^ and the (Hal3^PD_H378A^)_2_/Cab3^PD^ trimer due to the higher stability (and low exchange susceptibility) of the Vhs3^PD^ homotrimer compared to its Hal3^PD^ counterpart. Although it could also be argued that incorporation of Vhs3^PD^ leads to formation of a complex with an inherently lower PPCDC activity, we consider this a highly unlikely possibility based on the finding that the different heterotrimeric complexes showed comparable levels of activity (Fig. 3b), and the previous observation that incorporation of Vhs3 into a heterotrimer of the full length proteins resulted in an increase in PPCDC activity compared to that seen for a Hal3/Cab3 heterotrimer^5^.

Importantly, the inactive Hal3^PD_H378A^/Vhs3^PD_H459A^/Cab3^PD^ mixture only showed PPCDC activity upon titration with Hal3^PD^, and at much lower levels than that seen upon titration of the Hal3^PD_H378A^/Cab3^PD^ mixture with the same protein (Fig. 7). This indicates that the Hal3^PD^/Vhs3^PD^/Cab3^PD^ hetero-trimer is less susceptible to subunit exchange, and supports the interpretation of the flavin release experiments that the single T_m_-value is associated with the dissociation of the (Hal3^PD^)_2_/Cab3^PD^ complex present in such mixtures. It is in fact very likely that the PPCDC activity observed upon titration of the Hal3^PD_H378A^/Vhs3^PD_H459A^/Cab3^PD^ mixture with Hal3^PD^ is due to exchange of (Hal3^PD_H378A^)_2_/Cab3^PD^ present in the mixture, although currently we are unable to demonstrate this experimentally.

## Discussion

Although the disparate and non-overlapping activities of the *S. cerevisiae* Hal3 and Vhs3 proteins—either as constituents of a unique heterotrimeric PPCDC involved in CoA biosynthesis, or as regulatory subunits of the Ppz1 phosphatase—clearly identifies them as moonlighting proteins, the lack of structural information on these proteins made it difficult to rationalize the molecular basis whereby these activities are differentiated. Based on previous studies that showed that the Vhs3–Ppz1 interaction has a much smaller physiological relevance *in vivo* than that of Hal3–Ppz1^6^,^7^, this study was initiated by an investigation of the latter complex. The evidence presented here that Hal3 and Ppz1 interacts with a 1:1 stoichiometry (i.e. that Hal3 inhibits Ppz1 as monomer), provided the starting point for studying the possible dynamic exchange of Hal3 and Vhs3 among the relevant protein complexes.

**Figure 4:**
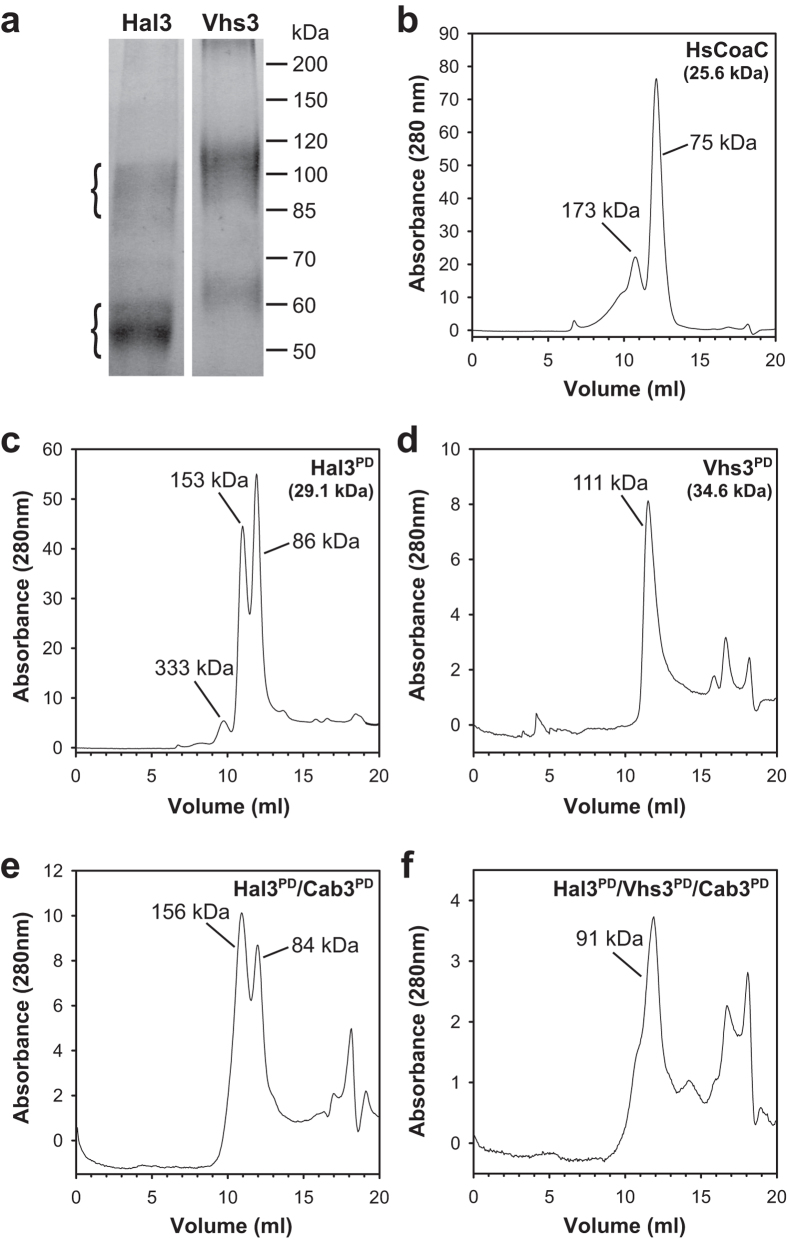
The PDs of Hal3, Vhs3 and Cab3 form trimers. (**a**) SDS-PAGE analyses (8% gel) of DSS-treated 6 × His-Hal3^PD^ (expected MWs: 58.2 kDa (dimer), 87.3 kDa (trimer)) and 6 × His-Vhs3^PD^ (expected MWs: 69.2 kDa (dimer), 103 kDa (trimer)). (**b–f**) Size exclusion chromatography (SEC) analyses of the purified PD proteins and mixtures: (**b**) HsCoaC; expected MWs: 25.6 kDa (monomer), 76.8 kDa (trimer) (**c**) Hal3^PD^; expected MWs: 29.1 kDa (monomer), 87.3 kDa (trimer), 153 kDa (hexamer), 349.2 kDa (dodecamer) (**d**) Vhs3^PD^; expected MWs: 34.6 kDa (monomer), 103 kDa (trimer) (**e**) Hal3^PD^/Cab3^PD^; expected MW: 26.2 kDa (average monomeric MW), 78.6 kDa (2 × Hal3^PD^/1 × Cab3 trimer); 157.2 kDa (4 × Hal3^PD^/2 × Cab3 hexamer) (**f**) Hal3^PD^/Vhs3^PD^/Cab3^PD^; expected MWs: 29 kDa (average monomeric MW), 87 kDa (1 × Hal3^PD^/1 × Vhs3^PD^/1 × Cab3 trimer).

In this study we investigated the activity, structure, thermal stability and exchange reactions of hetero-trimers of the Hal3, Vhs3 and Cab3 PDs in an attempt to gain a better understanding of the factors that affect the formation of the active PPCDC enzyme, and whether oligomerization dynamics could act as a putative regulatory mechanism. The PDs did prove to be suitable model constructs, as our data show that they are sufficient for PPCDC activity (Fig. 3b). Furthermore, Cab3^PD^ forms a strong interaction with Hal3^PD^/Vhs3^PD^ as evidenced by the fact that untagged Cab3^PD^ co-purified with 6 × His-tagged Hal3^PD^/Vhs3^PD^ (Fig. 3a). This observation, in combination with cross-linking, SEC and PPCDC activity results (Fig. 4), indicates that the PDs also contain the structural elements required for oligomerisation. Combined, these experiments suggest that study of the oligomeric nature of the PD complexes should be relevant and applicable to the PPCDC complex formed by the full-length proteins, and to the ability of Hal3 and Vhs3 to dissociate from it.

**Figure 5:**
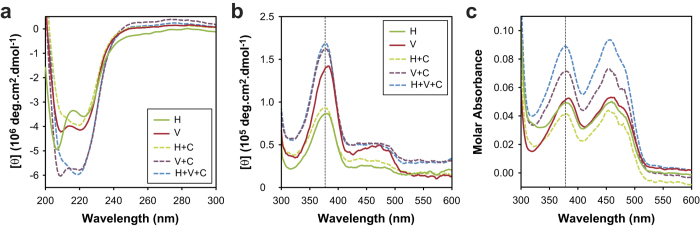
Vhs3 has a different secondary structure composition and mode of flavin binding. (**a**) CD spectra of Hal3^PD^ (H), Vhs3^PD^ (V), Hal3^PD^/Cab3^PD^ (H + C), Vhs3^PD^/Cab3^PD^ (V + C), and co-expressed Hal3^PD^/Vhs3^PD^/Cab3^PD^ (H + V + C) in the UV range, highlighting the differences and similarities in their secondary structures. (**b**) CD spectra of the same proteins and mixtures obtained in the visible range, showing the differences and similarities in the environments of their flavin cofactors. The vertical dotted line shows the absorbance maximum of all the proteins—with the exception of Vhs3^PD^—at 375 nm. (**c**) Absorbance spectra obtained simultaneously with the CD spectra in panel b. The legend is identical to that shown in (**b**).

**Figure 6:**
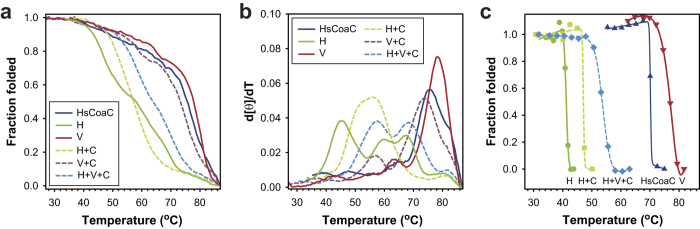
Heat-induced denaturation of the PD proteins is preceded by flavin release. (**a**) Melting curves of HsCoaC and Hal3^PD^ (H), Vhs3^PD^ (V), Hal3^PD^/Cab3^PD^ (H + C), Vhs3^PD^/Cab3^PD^ (V + C), and co-expressed Hal3^PD^/Vhs3^PD^/Cab3^PD^ (H + V + C) as determined by the changes in the proteins’ molar elipticity at 222 nm. The curves represent the fraction folded protein as a function of temperature. (**b**) First derivative plots of the melting curves shown in panel a, smoothed using the standard settings in SigmaPlot 11. (**c**) Plots of changes in the molar elipticity measured for the indicated proteins at 320 nm as a function of temperature; the curves represent the best fits of the Gibbs-Helmholtz equation to the data (see Methods for details).

In light of their high sequence homology (Vhs3^PD^ exhibit 62% sequence identity in comparison with Hal3^PD^), we were surprised to find that the Hal3 and Vhs3 PDs exhibit significant structural differences as observed by CD studies based on both secondary structure and mode of flavin binding (Fig. 5). The differences apparently also translate into significant differences in thermal stability, with homomeric Vhs3^PD^ exhibiting a T_m_ that is ~30 °C higher than that of the homomeric Hal3^PD^ (Fig. 6). Importantly, the heat-induced flavin-release experiments showed that the high thermal stability of Vhs3^PD^ makes it resistant to de-oligomerisation, as it only releases its flavin (bound at the subunit interfaces) upon denaturation of the whole complex. In contrast, the relative low stability of the Hal3^PD^ complexes seems to allow them to readily release monomeric subunits. Taken together, our findings indicate that Vhs3^PD^ preferentially exists as a trimer, while Hal3^PD^ is able to interchange between monomeric and higher order oligomeric forms.

**Figure 7:**
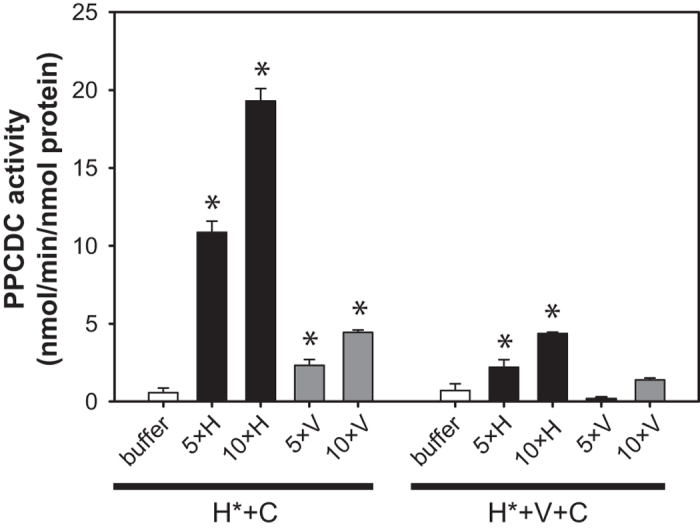
The heterotrimeric complexes exhibit differences in their ability to undergo subunit exchange. The heterotrimers Hal3^PD_H378A^/Cab3^PD^ (H* + C) and Hal3^PD_H378A^/Vhs3^PD_H459A^/Cab3^PD^ (H* + V* + C) were titrated with five- and 10-fold (relative to the monomer concentration of the inactive heterotrimer) of either Hal3^PD^ (H) or Vhs3^PD^ (V). The activities (reported as nmol/min/nmol protein) were adjusted to the monomer concentration of the inactive heterotrimers (i.e. excess Hal3^PD^ or Vhs3^PD^ added was not considered in the calculation). The data indicated the average of an experiment performed in triplicate; the error bars indicate the standard deviation. Values were compared to that of the inactive control (buffer) by one-way ANOVA followed by Dunnet’s post-test (*p < 0.05).

These differences between Hal3^PD^ and Vhs3^PD^ carry over to the heteromeric complexes of which they form part. First, the Hal3^PD^/Cab3^PD^ complex is significantly more stable than Hal3^PD^ on its own; however, like Hal3^PD^, it also readily allows dissociation of its constituent subunits (Fig. 6). This is further underscored by the experiments in which native Hal3^PD^ or Vhs3^PD^ was titrated to a mixture of Hal3^PD_H378A^/Cab3^PD^ (i.e., an inactive PPCDC complex) (Fig. 7). Addition of Hal3^PD^ causes a significant increase in activity, with much lower levels seen upon addition of Vhs3^PD^—clearly showing that Hal3^PD^ readily exchanges monomers between homomeric and heteromeric complexes, while this is not the case for Vhs3^PD^. In contrast, when the same experiment was conducted with the Hal3^PD_H378A^/Vhs3^PD_H459A^/Cab3^PD^ mixture the increase in activity was significantly reduced. This shows that addition of Vhs3^PD^ stabilised the complex, reducing its ability to exchange monomeric subunits. This analysis is supported by the thermal stability data, which shows that Hal3^PD^/Vhs3^PD^/Cab3^PD^ exhibits a higher T_m_ than Hal3^PD^/Cab3^PD^.

Overall, our results show that Hal3^PD^- and Vhs3^PD^-containing heteromeric complexes exhibit different stabilities, as well as disparate abilities to exchange their constituent monomers. While Hal3^PD^ can easily exchange from complexes containing only Hal3^PD^ and Cab3^PD^, the formation of the heterotrimeric Hal3^PD^/Vhs3^PD^/Cab3^PD^ complex by addition of Vhs3^PD^ makes it much more stable and less likely to show dynamic monomer exchange. It is important to note that the native expression of Hal3 is estimated to be 30-fold greater than Vhs3 (60 ppm versus ~2 ppm)^22^,^23^. It is tempting to speculate that these differences relate directly to the moonlighting roles of Hal3 and Vhs3, as indeed have been noted for other moonlighting proteins^24^. Our results suggest that Hal3 is more likely to act as Ppz1 inhibitor, while Vhs3 is more likely to be found as a stabilizing constituent of the heterotrimeric Hal3/Vhs3/Cab3 complex, ensuring that a certain amount of active PPCDC is always maintained in the cell. This hypothesis fits with the previous observation that, *in vivo*, Vhs3 is less effective than Hal3 in controlling Ppz1-mediated functions, as deduced from the phenotypic analysis of *HAL3* and *VHS3* deletion or overexpressing strains^6^,^7^. The difference in the relative abundance of the two proteins is compatible with Hal3 performing dual roles (as Ppz1 inhibitor and as component of the yeast PPCDC), while Vhs3 mainly forms part of the latter. It also agrees with our finding that in heterologous expression in *E. coli*, recombinant Cab3 co-expresses better with Hal3 than with Vhs3, which due to a lower stoichiometry is less likely to form a complex with only Cab3 (i.e. (Vhs3)_2_/Cab3) *in vivo*. The differentiation of Hal3 and Vhs3 might have conferred an evolutionary advantage that ensured that both proteins were maintained in the genome, even though they appear to exhibit similar functions *in vivo*, and largely equivalent properties *in vitro*^7^.

The structural basis for these differences between Hal3 and Vhs3 remain unclear. However, in spite of their high level of sequence similarity both proteins also contain large non-homologous insertions (Fig. 1c). It is possible that these regions play an important role in the stability of the enzymes, perhaps through their ability to form higher order oligomers (our SEC analysis data clearly shows that Vhs3^PD^ only forms trimers, while Hal3^PD^ and Hal3^PD^/Cab3^PD^ also forms apparent hexamers and dodecamers; see Fig. 4). Such a conclusion is supported by previous SEC analysis studies of Hal3 ∆312-350 (a construct in which its insertion is deleted), which showed that although deletion of the region did not affect homotrimerisation, the ability to form higher oligomeric structures was reduced^15^. A recent computational study indicated the general validity of this observation, as it was shown that insertions and deletions in homologous proteins profoundly affect their oligomeric states and complex stability^25^.

Recently, Hal3, Vhs3 and Cab3 were described as forming part of a large CoA synthesising protein complex (CoA-SPC) in yeast, which also involve the CoA biosynthesis enzymes Cab2, Cab4 and Cab5^18^. The existence of such a complex was previously proposed but not widely accepted as these authors also suggested that the complex biosynthesized CoA via an alternate pathway^26^,^27^. Olzhausen *et al.* systematically analysed interaction between the different yeast CoA biosynthesis enzymes, showing that Cab2, Cab3, Cab4 and Cab5 can interact with each other, supporting the existence of a large protein complex^18^. The authors also mapped the regions of these proteins that take part in these interactions, which suggested that Cab3 acts as a scaffold for the complex, primarily via interactions with its N-domain. Importantly, Cab3 homomerisation was also found to occur via the N-domain, which likely explains why we were unable to purify Cab3^PD^ on its own. These results indicate that the role of the N-domains of these proteins is to form and maintain the CoA-SPC *in vivo*.

Similarly, our previous studies on the functional mapping of the Hal3 domains showed that its N-terminal region is important to stabilize interaction between Hal3 and full-length Cab3, although the PPCDC domain was the minimal requirement for formation of the trimeric complexes containing these proteins^15^. The results of the current study provide further support for the PDs defining the primary nature of the Hal3/Vhs3 interaction with Cab3. Nonetheless, we cannot exclude that the presence of the N-terminal domain could impact on the analysis of the stabilities of the various complexes presented here. However, considering the high level of similarity between the N-and C-domains of especially Hal3 and Vhs3 (the N-domain of Vhs3 is 49% identical to that of Hal3 and 61% similar, and its C-domain 55% identical and 68% similar), we believe that while the inclusion of these domains may affect the absolute stability of the full length proteins, the relative stabilities should remain similar—and that the conclusions drawn from the study of the PD proteins should therefore also hold true for the native proteins. Confirmation of the binding interaction models on which these analyses are based must await determination of the crystal structures of the various complexes; however, in spite of significant efforts in this regard, we have thus far not been able to obtain such structures. Yet, it is clear that the existence of a large CoA-SPC makes the structural and biophysical properties of Hal3, Vhs3 and the heteromeric PPCDC oligomers of great interest as this likely plays a crucial role in the assembly and function of the CoA-SPC complex, and may increase the regulatory complexity of the moonlighting activities of Hal3 and Vhs3 even further.

In conclusion, in this study we demonstrated that the PDs of Hal3, Vhs3 and Cab3 are the minimum and sufficient requirement for these proteins to show PPCDC activity by forming active heterotrimeric complexes. Moreover, we show that Hal3 interacts with Ppz1 with a 1:1 ratio and that Hal3’s structural characteristics allow it to exist as a monomer that can readily be exchanged between higher order complexes. In contrast, Vhs3’s structure causes it to form stable complexes that are more resistant to monomer exchange. These findings provide the first indication of the molecular basis for differentiation between the homologous Hal3 and Vhs3 proteins, and give insight into a possible mechanism for the physiological regulation of their respective moonlighting activities. Future studies will have to address how such regulation occurs *in vivo*, including the impact of synchronized translation to ensure the simultaneous production of the constituent parts of the heteromeric complexes to ensure their preferential formation when required.

## Methods

### Plasmid construction

The PDs of Vhs3 (residues 288–577) and Cab3 (residues 305–508) were identified by comparison to HsCoaC, the human PPCDC (Fig. 1c). The corresponding sections of the respective genes were subsequently amplified by PCR using the previously described pGEX6P-1 vectors^5^ as templates with the oligonucleotides 5′-Vhs3CoaC, 3′-Vhs3CoaC, 5′-Ykl088wCoaC and 3′-Ykl088wCoaC (Supplementary Table S1). These oligonucleotides introduce NdeI and XhoI restriction sites which were used to subclone the obtained PCR products into the corresponding restriction sites of the kanamycin resistant expression plasmid pET28a (Novagen), to yield pET28a-Vhs3^PD^ and pET28a-Cab3^PD^ (Supplementary Table S2). The vector pET28-Hal3^PD^ was constructed previously in an analogous manner^15^. The plasmids pET28a-Hal3^PD_H378A^ and pET28a-Vhs3^PD_H459A^ were constructed similarly, with the previously described pGEX6P1-Hal3_H378A and pGEX6P1-Vhs3_H459A as templates^5^.

The PDs were also cloned into the ampicillin resistant co-expression vector pETDuet-1 (Novagen), which allows for the simultaneous expression of two proteins: the first with an N-terminal 6 × His fusion tag, and the second as an untagged protein. Cab3^PD^ was subcloned from pET28a-Cab3^PD^ into the second multiple cloning site (MCS) of the vector using the NdeI/XhoI restriction sites. For subcloning of Hal3^PD^, Hal3^PD_H378A^, Vhs3^PD^ and Vhs3^PD_H459A^ into the first MCS, these genes were first subcloned from the relevant pET28a plasmids into the entry vector pENTR4T using the restriction sites NcoI and XhoI, and subsequently subcloned into the NcoI/BsrGI restriction sites of the first MCS of pETDuet-1. In this manner, the co-expression plasmids pETDuet-1_Hal3^PD^_Cab3^PD^, pETDuet-1_Vhs3^PD^_Cab3^PD^ and pETDuet-1_Hal3^PD_H378A^_Cab3^PD^ were obtained.

Plasmids pGEX6P1-Ppz1^Cter^ and pGEX6P1-Hal3, which allows for the expression of the GST-fusions of Ppz1 (Δ1–344) (carboxyl terminal domain; Ppz1^Cter^) and the entire Hal3, respectively, were described previously^7^. Co-expression of Ppz1^Cter^ N-terminally fused to a 6 × His tag and the entire Hal3 was accomplished by cloning a Ppz1Cter fragment amplified by PCR with oligonucleotides pDuet_PPZ1^Cter^-5a and pDuet_Ppz1_T1-3 in the first MCS of pETDuet-1 (AscI/HindIII digestion), followed by subcloning the Hal3 fragment amplified with oligonucleotides pDuet_Hal3_5 and pDuet_Hal3_3, into the BglII/XhoI sites of the second MCS.

### Recombinant protein expression and purification

Expression was performed in chemically transformed *E. coli* BL21 (DE3) cells grown in Luria Bertani (LB) broth supplemented with 30 mg/L kanamycin and/or 100 mg/L ampicilin as required. Expression and purification of GST-Ppz1^Cter^ and GST-Hal3 was carried out as previously described^5^, except that in both cases induction was carried out with 0.1 mM IPTG, followed by overnight expression at 21 °C. Co-expression and purification of 6 × His-Ppz1^Cter^ and Hal3 was carried out in 1 L LB cultures, which was induced with 0.1 mM IPTG at an OD_600_ ~0.8–1.0, followed by overnight expression at 18 °C. Cells were collected by centrifugation at 3600 × g for 1 h at 4 °C and pellets resuspended in 30 mL of binding buffer (50 mM Tris-HCl, pH 7.5, 300 mM NaCl, 10 mM imidazole) supplemented with 0.1% Triton X-100 and protease inhibitor cocktail (Mini EDTA-free, Roche). The suspension was sonicated and centrifuged at 4 °C (12400 × g for 30-40 minutes), followed by Ni^2+^-affinity chromatography (His-Trap column, GE Healthcare) using an ÄKTA Purifier 100 system (GE Healthcare). After loading, the column was sequentially washed with binding buffer supplemented with 30, 60, and 80 mM imidazole (10 mL each, 1 mL/min), after which the protein complex was eluted with the same buffer containing 500 mM imidazole (1 mL/min). The fractions were adjusted to 5 mM EDTA and 2 mM DTT and stored at 4 °C. For size exclusion chromatography (SEC) the relevant fractions were pooled and concentrated to a volume of 500 μL by centrifugation in a Vivaspin 6 (50 MW cut-off, Sartorius) device. SEC was carried out by using a Superdex 200 HR 10/30 column (GE Healthcare) at a flow rate of 0.5 mL/min (1 mL fractions) with a buffer consisting of 50 mM Tris-HCl (pH 7.5), 270 mM NaCl, 2 mM DTT and 2 mM EDTA.

Large scale expression of 6 × His-Hal3^PD^ was performed as previously described^15^, while 6 × His-Vhs3^PD^ was successfully purified by using an identical protocol. Briefly, these proteins were expressed in 500 mL LB, induced with 0.2 mM IPTG at OD_600_ ~ 0.6 and allowed to grow overnight at 25 °C. A mixture of 6 × His-Hal3^PD^ and Cab3^PD^, and of 6 × His-Vhs3^PD^ and Cab3^PD^, were expressed from the respective pETDuet-1 plasmids by induction of the cultures with 0.05 mM IPTG at OD_600_ ~0.6, followed by overnight incubation at 37 °C. A mixture of 6 × His-Hal3^PD^, 6 × His-Vhs3^PD^ and Cab3^PD^ was expressed from BL21 (DE3) cells co-transformed with pETDuet-1_Hal3^PD^_Cab3^PD^ and pET28a-Vhs3^PD^ and expressed using the same conditions as for the pETDuet-1 plasmids. Mixtures of the catalytically inactivated mutants 6 × His-Hal3^PD_H378A^/Cab3^PD^, 6 × His-Hal3^PD_H378A^/Vhs3^PD^/Cab3^PD^ and 6 × His-Hal3^PD_H378A^/Vhs3^PD_H459A^/Cab3^PD^ were obtained similarly. 6 × His-HsCoaC was expressed and purified as previously described^28^.

Successfully expressed PD proteins were purified on an ÄKTAprime system (GE Healthcare), using Ni^2+^-affinity chromatography with HiTrapFF or HiTrap chelating columns (GE Healthcare) according to the manufacturer’s directions. Briefly, cell pellets collected from the 500 mL expressions were re-suspended in binding buffer (10 mL buffer/gram of cells), sonicated and subsequently centrifuged to remove cellular debris. The obtained cell extracts were loaded onto the column, followed by washing with binding buffer and 15% elution buffer to elute non-specifically bound proteins, and subsequent elution of the protein of interest in 100% elution buffer. The imidazole present after elution was removed by buffer exchange to gel-filtration buffer using a HiTrap desalting column (GE Healthcare) on the ÄKTAprime system.

Several different buffer systems were used for purification, each consisting of a binding buffer and gel-filtration buffer used for final buffer exchange. In all cases the protein was eluted with 500 mM imidazole added to the binding buffer. “Low Tris” buffer (20 mM Tris-HCl, pH 7.9, 500 mM NaCl, 0.05% (w/v) NaN_3_), and gel-filtration to 25 mM Tris-HCl, pH 8.0, 5 mM MgCl_2_ was used for the general purification of Hal3^PD^ and Vhs3^PD^. A phosphate buffer (20 mM phosphate, pH 7.4, 500 mM NaCl) and gel-filtration to 25 mM phosphate, pH 7.4, 5 mM MgCl_2_ was used for general purification of the co-expressed proteins, which tends to precipitate upon purification with Low Tris buffers. Finally, “High Tris” buffer (50 mM Tris-HCl, pH 8, 500 mM NaCl, 10% glycerol, 0.05% (w/v) NaN_3_) and gel-filtration to 50 mM Tris-HCl, pH 8, 150 mM NaCl, 10% glycerol was used for the CD spectroscopic studies due to increased protein stability.

### Protein concentration determination

Protein concentration was determined by means of the Bradford assay^29^ modified for use in a 96-well plate, and by a microbiuret assay^30^ for the circular dichroism studies, due to the reduced sensitivity of this assay to differences in protein sequence. For GST-Ppz1^Cter^ and Hal3 binding experiments, the amounts of the specific recombinant proteins was determined by SDS-PAGE followed by Coomassie staining and integration of the intensity of the relevant bands, using known amounts of bovine serum albumin as standards.

### Protein-protein interaction determination by pull-down

Aliquots of glutathione agarose beads containing ~6 μg of bound GST-Ppz1^Cter^ were incubated with increasing amounts of full length Hal3, prepared by digestion of recombinant GST-Hal3 with PreScission protease, for 1 h at 24 °C. The beads were recovered by centrifugation at 750 × *g* for 5 min at 4 °C and subsequently washed 3 times with 200 μL of washing buffer (50 mM Tris-HCl pH 7.5, 150 mM NaCl, 10% glycerol, 2 mM DTT, 0.5 mM PMSF and protease inhibitor cocktail (Roche). Finally, the beads were resuspended in 100 μL of washing buffer and 33 μL of 4 × Laemmli buffer was added. The mixture was boiled for 5 min, centrifuged at 750 × *g* for 2 min at 4 °C and the supernatant recovered for SDS-PAGE analysis.

### In vitro PPCDC assays

PPCDC assays were performed as previously described, using HsCoaC as positive control^5^. This assay couples the formation of the 4′-phosphopantetheine product to *E. coli* phosphopantetheine adenylyltransferase (*Ec*PPAT), the next enzyme in the CoA pathway. The assay links the pyrophosphate released by this enzyme to the oxidation of NADH, which is followed spectrophotometrically. Briefly, proteins (either individually or in mixtures, with a total concentration of 60 nM), were added to the individual wells of a 96-well plate, followed by initiation of the reaction by addition of the rest of the assay mixture (60 μL pyrophosphate reagent (Sigma-Aldrich), 0.5 mM *P*PC^31^, 2 mM ATP, 1 mM DTT, 10 mM MgCl_2_, 20 mM KCl and ~1.5 μM 6 × His-*Ec*PPAT^32^ in 50 mM Tris-HCl, pH 7.6). The final reaction volume was 150 μL. The reaction progress was followed at 340 nm and 37 °C using a microplate-based UV spectrophotometer (Varioskan, ThermoLabsystems).

### Monomer exchange experiments

Monomer exchange experiments were performed by evaluating the activity of 120 mM of catalytically inactive heteromers after incubation for 10 min at 37 °C, in the presence of excess Hal3^PD^ or Vhs3^PD^.

### PD size exclusion chromatography and cross-linking experiments

Size exclusion chromatography of PD complexes was performed on an ÄKTApurifier with a Superose 12 column (Amersham), with 50 mM phosphate (pH 7.0), 150 mM NaCl as eluant at a flow rate of 0.4 mL/min. The column was calibrated with suitable protein standards in a range from 29 kDa to 700 kDa (Sigma kit). Approximately 100 μL of the respective proteins purified in phosphate buffers were loaded onto the column.

Cross-linking experiments were performed using disuccinimydyl suberate (DSS) (Sigma) as cross-linking reagent, using a method derived from previously published procedures^33^,^34^. The DSS was dissolved in DMSO as a 24 mM stock solution, and added to protein samples (1:100 and 1:50 v/v for Hal3^PD^ and Vhs3^PD^ respectively) in 50 μL reaction volumes, followed by incubation at 37 °C for 15 and 40 min for Hal3^PD^ and Vhs3^PD^ respectively. The cross-linking reactions were quenched by addition of 10% of 1 M Tris-HCl, pH 8, followed by SDS-PAGE analyses using 8% gels.

### Circular dichroism (CD) studies

Several different types of studies were performed by using CD by using a Chirascan spectrapolarimeter (Photophysics) with protein purified using the described HT buffers.

#### Far-UV CD

Far-UV CD spectra were obtained between 200 and 300 nm (using 1 nm increments) to obtain information about the respective proteins’ secondary structures. Samples contained 0.2 mg/mL protein and were analysed at 25 °C using a 0.05 cm cuvette. Each spectrum represents an average of at least three scans, and was smoothed using the Chirascan software. The obtained raw data (ellipticity (θ) in millidegrees) were converted to molar ellipticity ([θ]) with the units of deg. cm^2^.dmol^-1^ using the equation:


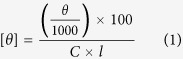


where C is the concentration in M and *l* refers to the cuvette path length in cm. For the co-expressed Cab3^PD^-containing mixtures the protein concentration was calculated by using a molecular weight estimated from the approximate ratios of the different proteins obtained as assessed by SDS-PAGE. For the protein mixtures the approximate ratios were Hal3^PD^/Cab3^PD^ (2:1), Vhs3^PD^/Cab3^PD^ (2:1) and Hal3^PD^/Vhs3^PD^/Cab3^PD^ (2:1:1).

#### Visible CD and absorbance spectra

CD spectra in the visible region were collected between 300 and 700 nm (using 1 nm increments) to gain information about the environment of the flavin cofactor. The CD and absorbance of protein samples (0.5 mg/mL protein) was measured simultaneously at 25 °C using a 0.4 cm cuvette. Each spectrum represents the average of at least three scans, and was smoothed using the Chirascan software. The obtained data was converted to [θ] as above.

#### Determination of protein melting curves based on protein unfolding

Protein melting curves were determined by measuring the change in the CD signal at 222 nm as a function of temperature of samples (0.2 mg/mL protein) contained in a 0.05 cm cuvette. Changes were measured between 35 °C and 94 °C using a heating rate of 2 °C/min (temperature tolerance was maintained at 0.2 °C). The raw data was converted to fraction of folded protein using equation 2:


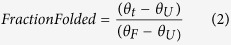


where θ_t_ is the ellipticity at any temperature, θ_F_ is the ellipticity of the fully folded protein and θ_U_ is the ellipticity of the fully unfolded protein^20^. We assumed that the initially obtained absorbance values were those of the fully folded protein, and that the values obtained at ~90 °C represented the CD absorbance of the fully unfolded protein. The first derivative data was smoothed using the standard settings of SigmaPlot 11 (Systat Software), and the absorbance data was normalized to have values between 0 and 1 for ease of comparison.

#### Determination of protein melting curves based on flavin release

Protein melting curves were also obtained by studying the changes in the CD spectra of the flavin cofactor of samples (0.5 mg/mL protein contained in a 0.4 cm cuvette) as a function of temperature. Spectra (300–500 nm) were measured at 1 or 2.5 °C increments (temperature tolerance was maintained at 0.2 °C), with a setting time of 30 s. The obtained spectra were converted to melting curves by selecting the CD values at 320 nm (where a large change in CD signal with temperature was observed). The obtained curves were converted to fraction folded protein using equation 1. The melting temperatures (T_m_) were subsequently determined by fitting the resulting curves simultaneously to equations 3, 4 and 5 (the Gibbs-Helmholtz equation) using SigmaPlot 11.0 (Systat Software):


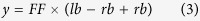



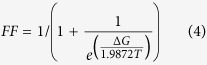



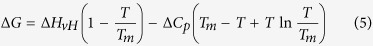


where *lb* and *rb* are the equations for the best linear fits to the left and right baselines of the curves respectively, FF refers to fraction folded, ΔG is the change in free energy of unfolding, ΔH_vH_ is the van’t Hoff enthalpy, T is the temperature at any point and ΔC_p_ is the change in heat capacity. The following parameter constraints were used for the curve fit: van’t Hoff enthalpy (ΔH_vH_) > 0; T_m_ > 0 and ΔC_p_ > 1000 J/K. This equation was chosen in preference to equations derived for homo- and heterotrimers^20^,^35^, due to the observation of a single transition state based on the release of the flavin cofactor.

## Additional Information

**How to cite this article**: Abrie, J.A. *et al.* Complex stability and dynamic subunit interchange modulates the disparate activities of the yeast moonlighting proteins Hal3 and Vhs3. *Sci. Rep.* **5**, 15774; doi: 10.1038/srep15774 (2015).

## Supplementary Material

Supplementary Information

## Figures and Tables

**Table 1:** T_m_-values determined based on the heat-induced changes in the PD proteins’ secondary structures and the heat-induced release of their flavin cofactors.

Protein/Mixture	T_m_ based onsecondary structurechanges^a^(CD at 222 nm)			T_m_ based onflavin release^b^(CD at 320 nm)
	T_m_^1^	T_m_^2^	T_m_^3^	T_m_
HsCoaC	65.6	**75.5**	–	70.2
Hal3^PD^	**45.0**	60.1	67.6	41.2
Vhs3^PD^	63.2	**78.4**	–	78.1
Hal3^PD^/Cab3^PD^	**55.8**	–	–	47.1
Vhs3^PD^/Cab3^PD^	56.8	**73.5**	–	nd^c^
Hal3^PD^/Vhs3^PD^/Cab3^PD^	57.7	**68.5**	–	53.8

^a^Determined from the maxima observed in the first derivative plots (Fig. 6b) of the heat-induced melt curves shown in Fig. 6a. The value in bold indicates the value that is most likely physiologically relevant (see text for details).

^b^Determined by fitting the data shown in the plots in Fig. 6c to the Gibbs-Helmholtz equation (see Experimental for details).

^c^nd, not determined.
