# Supplementary material for: Complex stability and dynamic subunit interchange modulates the disparate activities of the yeast moonlighting proteins Hal3 and Vhs3
Source: Sci Rep. 2015 Oct 30;5:15774. doi: 10.1038/srep15774 (PMC4626798; doi:10.1038/srep15774)
Supplement: Supplementary Information [file srep15774-s1.pdf]

Complex stability and dynamic subunit interchange modulates the disparate activities of the yeast moonlighting proteins Hal3 and Vhs3

J. Albert Abrie[1], Cristina Molero[2], Joaquín Ariño[2] and Erick Strauss[1*]

[1]Department of Biochemistry, Stellenbosch University, South Africa
[2]Institut de Biotecnologia i Biomedicina and Departament de Bioquímica i Biologia Molecular, Universitat Autònoma de Barcelona, Spain

SUPPLEMENTARY INFORMATION

Supplementary Table S1: List of oligonucleotides

Oligonucleotide name	Utilization	Sequence
5’-Vhs3CoaC	Construction of pET28a-Vhs3-PD	5’-GGATCCAAGGCTTCATATGGATGACGGCAAGC-3’
3’-Vhs3CoaC	Construction of pET28a-Vhs3-PD	5’-CATCCTCTTCTTCCTCGAGTCACGCAGAAACATCG-3’
5’-Ykl088wCoaC	Construction of pET28a-Cab3-PD	5’-GAGTTCTTTCAGCATATGGACGACAAGAAATTC-3’
3’-Ykl088wCoaC	Construction of pET28a-Cab3-PD	5’-CTTGCTCTTTATCTCGAGTTTCTCAGTCCCTAGCTTTC-3’
pDuet_PPZ1^{Cter}-5a	Construction of pET-Duet1 Ppz1^{Cter}_Hal3 (MCS 1)	5'-TTGGCGCGCCTGATAATGCAATCTTCCGGAA-3'
pDuet_PPZ1_T1-3	Construction of pET-Duet1 Ppz1^{Cter}_Hal3 (MCS 1)	5'-GGGAAGCTTCAATACACACGCGAAG-3'
pDuet_Hal3_5	Construction of pET-Duet1 Ppz1^{Cter}_Hal3 (MCS 2)	5'-GCAAGATCTGACTGCCGTCGCCTCTACT-3
pDuet_Hal3_3	Construction of pET-Duet1 Ppz1^{Cter}_Hal3 (MCS 2)	5'-CCGCTCGAGTTATTGATGCTTATCTATT-3

Supplementary Table S2: List of plasmids

Plasmid	Utilization	Origin/ Reference
pENTR4T-Hal3^{PD}	Construction of pETDuet-1_Hal3^{PD}_Cab3^{PD}	This work
pENTR4T-Vhs3^{PD}	Construction of pETDuet-1_Vhs3^{PD}_Cab3^{PD}	This work
pET28a-Cab3^{PD}	Attempted expression of 6xHis-Cab3^{PD}	This work
pET28a-Hal3^{PD}	Expression of 6×His-Hal3^{PD}	Ref. 1
pET28a-Vhs3^{PD}	Expression of 6×His-Vhs3^{PD}	This work
pET28a-Vhs3^{PD_H459A}	Expression of 6×His-Vhs3^{PD_H459A}	This work
pETDuet-1_Cab3^{PD} (MCS2)	Construction of pETDuet-1 plasmids	This work
pETDuet-1_Hal3^{PD}_Cab3^{PD}	Co-expression of 6×His-Hal3^{PD} and Cab3^{PD}	This work
pETDuet-1_Hal3^{PD_H378A}_Cab3^{PD}	Expression of 6×His-Hal3^{PD_H378A} and Cab3^{PD}	This work
pETDuet-1_Vhs3^{PD}_Cab3^{PD}	Co-expression of 6×His-Vhs3^{PD} and Cab3^{PD}	This work
pPROEX-Hta-HsCoaC	Expression of 6×His-HsCoaC	Ref. 2
pGEX6P1-Hal3	Expression of GST-Hal3/Construction of pET28a-Hal3^{PD}	Ref. 3
pGEX6P1-Vhs3	Construction of pET28a-Vhs3^{PD}	Ref. 3
pGEX6P1-Ykl088w	Construction of pET28a-Cab3^{PD}	Ref. 4
pGEX6P1-Hal3_H378A	Construction of pETDuet-1_Hal3^{PD_H378A}_Cab3^{PD}	Ref. 4
pGEX6P1-Vhs3_H459A	Construction of pET28a-Vhs3^{PD_H459A}	Ref. 4
pGEX6P1-Ppz1^{Cter}	Expression of GST-Ppz1(Δ1–344)	Ref. 3
pETDuet-1_Ppz1^{Cter}_Hal3	Co-expression of 6×His-Ppz1^{Cter} and Hal3	This work

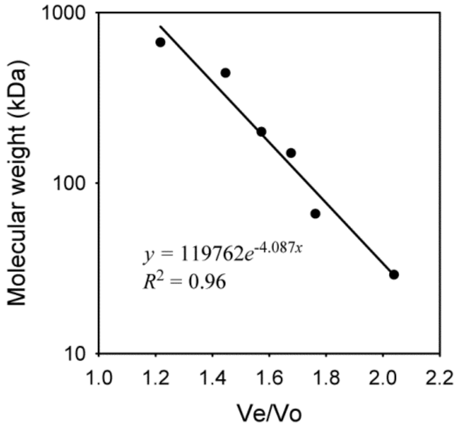

Supplementary Fig. S1. SEC calibration curve. Calibration curve used to determine the size of the protein and complex peaks in the SEC elution profiles shown in Figure 4b–f.

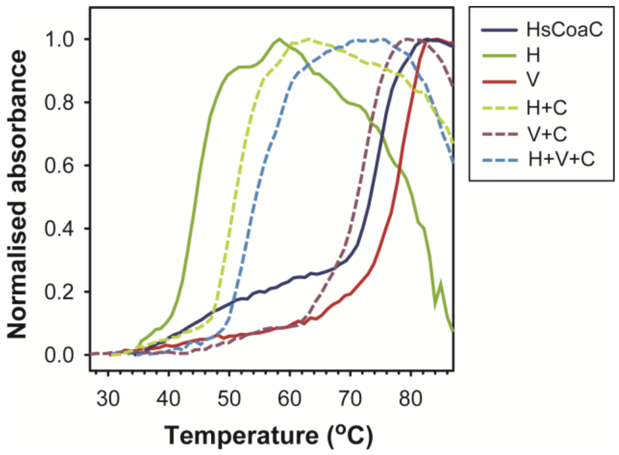

Supplementary Fig. S2. Heat-induced changes in the PD proteins' absorbance at 222 nm. Normalised absorbance data collected simultaneously with the circular dichroism data shown in Figure 6a.

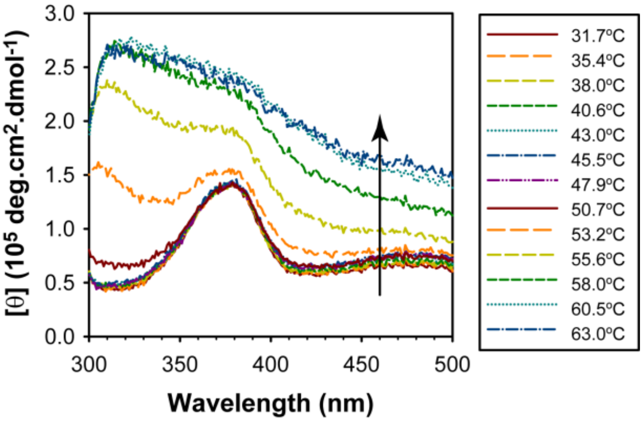

Supplementary Fig. S3. Typical changes in the visible CD spectra of the PD proteins observed as a function of temperature. CD spectra of the $Hal3^{PD}/Vhs3^{PD}/Cab3^{PD}$ complex obtained at increasing temperatures (as indicated by the arrow), with the legend next to the figure indicating the temperature at which the respective spectra were obtained.

References

1 Abrie, J. A., González, A, Strauss, E & Ariño, J. Functional mapping of the disparate activities of the yeast moonlighting protein Hal3. *Biochem. J.* **442**, 357-368 (2012).

2 Daugherty, M., Polanuyer, B., Farrell, M., Scholle, M., Lykidis, A., de Crecy-Lagard, V. & Osterman, A. Complete reconstitution of the human coenzyme A biosynthetic pathway via comparative genomics. *J. Biol. Chem.* **277**, 21431-21431 (2002).

3 Ruiz, A., Munoz, I., Serrano, R., Gonzalez, A., Simon, E. & Arino, J. Functional Characterization of the *Saccharomyces cerevisiae* VHS3 Gene. *J. Biol. Chem.* **279**, 34421-34421 (2004).

4 Ruiz, A., Gonzalez, A., Munoz, I., Serrano, R., Abrie, J. A., Strauss, E. & Arino, J. Moonlighting proteins Hal3 and Vhs3 form a heteromeric PPCDC with Ykl088w in yeast CoA biosynthesis. *Nat. Chem. Biol.* **5**, 920-928 (2009).
